# On the association between earlobe crease and the carotid intima-media thickness: A population-based study

**DOI:** 10.1016/j.heliyon.2019.e01556

**Published:** 2019-04-23

**Authors:** Oscar H. Del Brutto, Robertino M. Mera, Aldo F. Costa, Pablo R. Castillo, Gautam Matcha

**Affiliations:** a School of Medicine, Universidad Espíritu Santo – Ecuador, Guayaquil, Ecuador; b Department of Epidemiology, Gilead Sciences, Inc., Foster City, CA, USA; c Sleep Disorders Center, Mayo Clinic School of Medicine, Jacksonville, Fl, USA; d Internal Medicine Department, Mayo Clinic School of Medicine, Jacksonville, Fl, USA

**Keywords:** Anatomy, Epidemiology

## Abstract

The earlobe crease (ELC) has been linked to coronary artery disease, but there is limited information on the association between ELC and extracranial atherosclerosis. Using the Atahualpa Project cohort, we aimed to assess the association between ELC and increased carotid intima-media thickness (cIMT). Atahualpa residents aged ≥40 years underwent visual inspection of both earlobes to evaluate ELC presence, and ultrasound examinations of carotid arteries to calculate the cIMT. The association between both variables was assessed by logistic regression and predictive models, after adjusting for relevant confounders. Mean age of 570 enrolled individuals was 61.5 ± 12.4 years (58% women). ELC was present in 221 (39%) participants. The mean cIMT was 0.85 ± 0.19 mm, with 81 individuals (14%) having an increased cIMT (>1 mm). Univariate logistic regression showed a significant association between ELC presence and increased cIMT (OR: 1.67; 95% C.I.: 1.04–2.69), which disappeared when age (OR: 1.09; 95% C.I.: 0.65–1.85) and other covariables (OR: 1.06; 95% C.I.: 0.62–1.84) were added to the model. Predictive cIMT margins did not differ according to ELC presence or absence, with participants stratified in quartiles of age. This study shows that the effect of the increase in cIMT in subjects with ELC is related to aging.

## 1 Introduction

Earlobe crease (ELC), also known as Frank's sign, is a wrinkle extending from the tragus to the outer border of the earlobe [1]. This condition has been linked to coronary artery disease and other vascular conditions related to atherosclerosis [2]. However, pathogenetic mechanisms attempting to explain the association between ELC and atherosclerosis are not fully understood. It is possible that abnormalities in collagen metabolism – involved in atherosclerosis progression – also occur in the skin [3]. It is also possible that the ELC might be genetically determined, which might explain its different correlates with atherosclerosis according to race/ethnicity [4]. Information on the association between ELC and the carotid intima-media thickness (cIMT) – a surrogate of extracranial atherosclerosis – is limited [5, 6, 7, 8, 9]. Using the Atahualpa Project cohort, we aimed to assess this association in community-dwelling adults.

## 2 Methods

The study was conducted in Atahualpa, a rural Ecuadorian village. The population is homogeneous regarding race/ethnicity, living characteristics and dietary habits [10]. Atahualpa residents aged ≥40 years identified by means of door-to-door surveys who signed the informed consent were enrolled. The Institutional Review Board of Hospital-Clínica Kennedy, Guayaquil (FWA 00006867) approved the study. Participants underwent visual inspection of both earlobes to evaluate ELC presence, and ultrasound examinations of carotid arteries to calculate the cIMT. The association between both variables was assessed by logistic regression and predictive models, after adjusting for relevant confounders (see below).

**Fig. 1:**
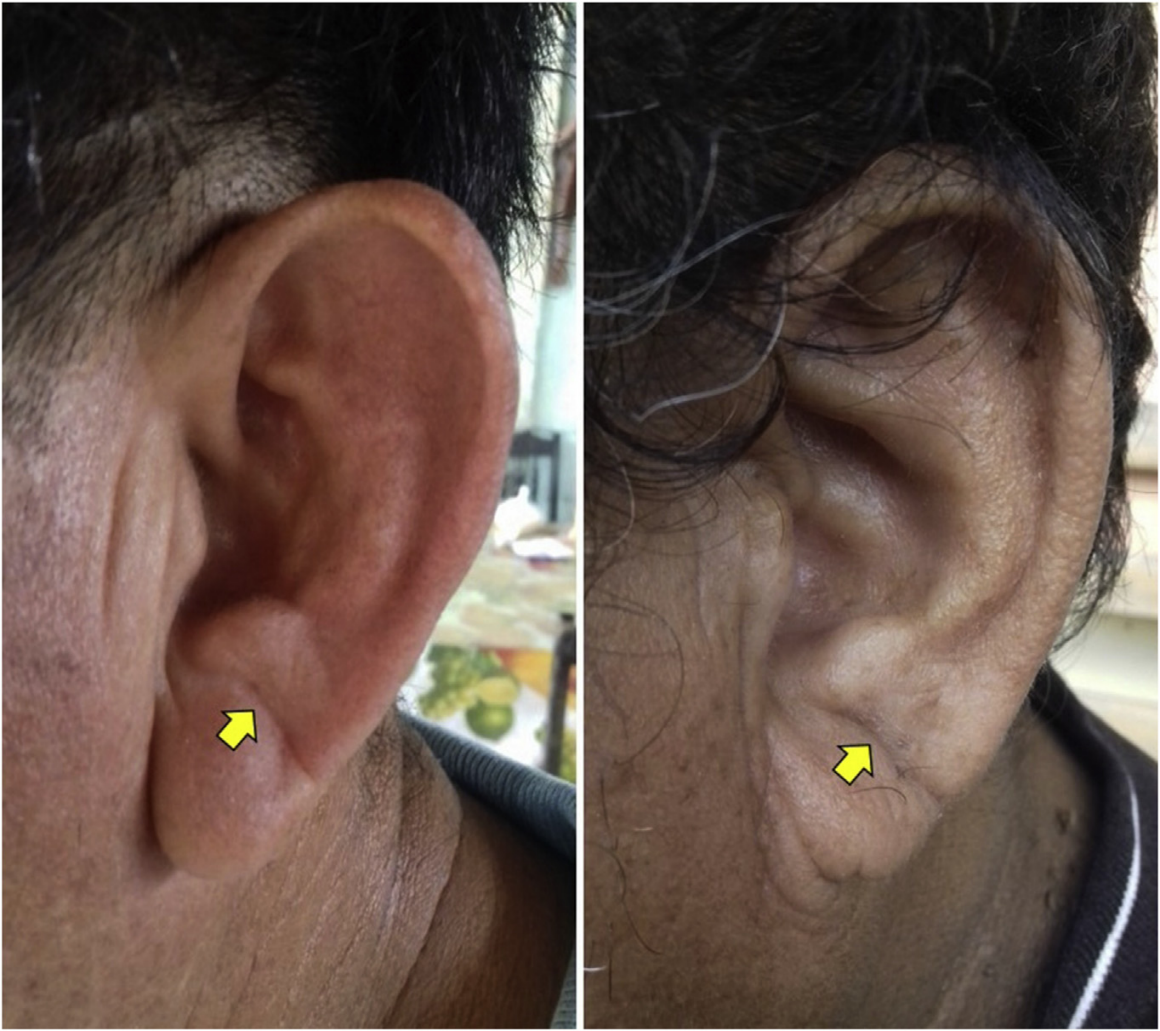
Earlobe crease in two study participants appearing as a wrinkle extending from the tragus to the outer border of the earlobe (arrows).

Both earlobes were examined with the subject in the sitting position. An ELC was considered to be present when the individual has a wrinkle extending from the tragus to the outer border of the earlobe (Fig. 1). Subjects with creases related to earrings and those who have distorted earlobe anatomy were excluded. Two investigators independently assessed the earlobes. Inter-rater agreement was excellent for ELC presence (kappa = 0.95). Discrepancies were resolved by consensus.

Carotid B-mode ultrasounds were performed by the use of a Terason Smart 3300 NexGen ultrasound scanner (Teratech Corporation, Burlington, MA, USA) and a 4–15 MHz linear probe. Assessment of the cIMT consisted of scanning each of the carotid arteries in three segments, including, the near and far wall of the segment extending from 10 to 20 mm proximal to the tip of the flow divider into the common carotid artery (CCA); the near and far wall of the carotid bifurcation beginning at the tip of the flow divider and extending 10 mm proximal to the flow divider tip; and 3) the near and far wall of the proximal 10 mm of the internal carotid artery (ICA). The cIMT was calculated as the mean of these 12 sites (six left and six right), and was considered increased if >1 mm [11].

Covariables investigated included demographics and cardiovascular risk factors (smoking status, physical activity, diet, the body mass index, blood pressure, fasting glucose, and total cholesterol blood levels), which were assessed by means of interviews and procedures previously described in the Atahualpa Project, using criteria proposed by the American Heart Association [10].

Data analyses are carried out by using STATA version 15 (College Station, TX, USA). In univariate analyses, continuous variables were compared by linear models and categorical variables by *x*^2^ or Fisher exact test as appropriate. A multivariate logistic regression model was fitted to evaluate whether ELC presence was associated with an abnormal cIMT (dependent variable), after adjusting for demographics and cardiovascular risk factors. In view of the known increased prevalence of ELC and higher cIMT values with advancing age, a predictive model was then fitted to assess the association of the average cIMT with ELC presence (uni- or bilateral), with participants stratified in quartiles of age.

## 3 Results

Of 863 individuals aged ≥40 years enrolled up to June 2017, 693 were active at the time of this study. The others had died, moved out of the village or declined consent. Carotid ultrasound examinations were performed in 594 of 693 individuals (86%); the remaining subjects declined to participate. Four additional individuals were excluded due to motion artifacts precluding evaluation of the cIMT, and 20 because of earlobe deformities impeding characterization of ELC.

ELC was present in 221 (39%) participants (unilateral in 111 and bilateral in 110). The mean cIMT was 0.85 ± 0.19 mm (81 individuals [14%] had a cIMT >1 mm). Table 1 shows the characteristics of participants across categories of ELC and cIMT. As noticed, individuals with an ELC were older than those with absent ELC. On the other hand, subjects with an increased cIMT were older, more often men, hypertensive and diabetic than those with a normal cIMT.

**Table 1:** Characteristics of Atahualpa residents aged ≥40 years included in this study (univariate analyses).

		Total series (n = 570)	Earlobe crease			Carotid intima media thickness		
			Absent (n = 349)	Present (n = 221)	*p* value	Normal (n = 489)	Increased (n = 81)	*p* value
Age, years (mean ± SD)		61.5 ± 12.4	59 ± 12.3	65.4 ± 11.5	<0.001^a^	59.5 ± 11.5	73.2 ± 11.1	<0.001^a^
Women, n(%)		329 (58)	210 (60)	119 (54)	0.136	298 (61)	31 (38)	<0.001^a^
Current smokers, n(%)		18 (3)	11 (3)	7 (3)	0.992	15 (3)	3 (4)	0.731
Body mass index ≥30 kg/m^2^, n(%)		162 (28)	95 (27)	67 (30)	0.425	146 (30)	16 (20)	0.083
Poor physical activity, n(%)		37 (7)	23 (7)	14 (6)	0.904	28 (6)	9 (11)	0.115
Poor diet, n(%)		27 (5)	17 (5)	10 (5)	0.849	20 (4)	7 (9)	0.133
Blood pressure ≥140/90 mmHg, n(%)		187 (33)	105 (30)	82 (37)	0.082	137 (28)	50 (62)	<0.001^a^
Fasting glucose ≥126 mg/dL, n(%)		153 (27)	88 (25)	65 (29)	0.271	123 (25)	30 (37)	0.036^a^
Total cholesterol ≥240 mg/dL, n(%)		61 (11)	43 (12)	18 (8)	0.116	52 (11)	9 (11)	0.888

a Significant result.

**Fig. 2:**
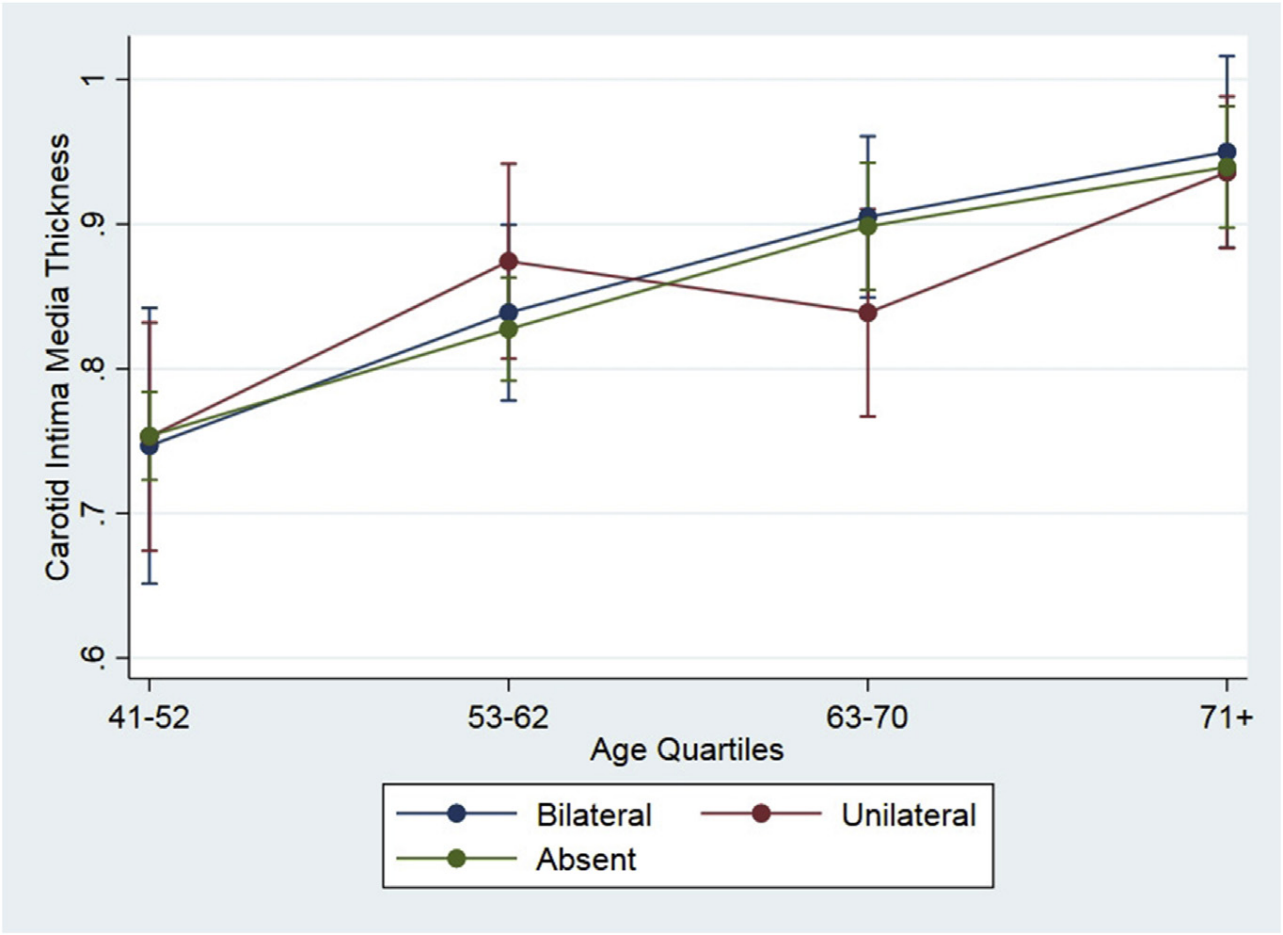
Graph plot showing predictive margins (with 95% C.I.) of cIMT according to the presence of uni- or bilateral ELC, or its absence, with participants stratified in quartiles of age. There is a parallel increase in cIMT margins according to increasing age, irrespective of ELC.

Univariate logistic regression showed a significant association between ELC presence and increased cIMT (OR: 1.67; 95% C.I.: 1.04–2.69; *p* = 0.033), which disappeared when age (OR: 1.09; 95% C.I.: 0.65–1.85; *p* = 0.730) and cardiovascular risk factors (OR: 1.06; 95% C.I.: 0.62–1.84; *p* = 0.821) were added to the model. In the latter, covariates remaining significant were age (*p* < 0.001), sex (*p* = 0.014) and high blood pressure (*p* = 0.003). Predictive margins of the cIMT did not differ according to ELC presence (uni- or bilateral) or absence, with participants stratified in quartiles of age (Fig. 2).

## 4 Discussion

This study shows a significant association between ELC presence and increased cIMT in univariate analysis. This association became non-significant when age and other covariables were added to regression models. Predictive models confirmed that all the effect of the change in the cIMT was because of aging and not because of ELC.

As noted, the literature reveals an apparently strong association between ELC and coronary artery disease. In a series of 1,000 individuals prospectively evaluated by a single author, a total of 373 had ELC, 275 of whom (74%) and coronary artery disease. Of the remaining 627 without ELC, only 101 (16%) had coronary artery disease (p < 0.001) [12]. These findings have been corroborated in subsequent studies, providing a robust epidemiological association between ELC and coronary artery disease [4, 13]. However, other studies have cast doubts on the strength of this association [14]. The same has occurred with studies attempting to assess the association between the ELC and other markers of atherosclerosis, including peripheral artery disease [15, 16].

Most of the studies trying to demonstrate an association between ELC and cIMT disclosed an apparent association between both variables [5, 6, 7, 8, 9]. However, the aforementioned studies were flawed by either biased selection of participants, a small sample size, limited assessment of the cIMT to a single artery wall, and different cutoffs used for defining an increased cIMT (Table 2). The present study included unbiased selection of participants and global calculation of the cIMT by assessing 12 different measurement of both carotid systems. These factors represent major strengths of our study. Potential limitations are the cross-sectional design and the fact that Atahualpa residents might not be representative of people living in other settings.

**Table 2:** Studies evaluating the association between the earlobe crease and the carotid intima media thickness.

Author, year (study design)	Number of participants	Artery investigated	Results
Çelik et al., 2007 (case control)	65 cases with ELC and 65 controls	CCA, at 1 cm of bifurcation.	cIMT higher in patients with ELC than in controls (0.88 ± 0.14 *vs*. 0.69 ± 0.14 mm; p < 0.001)
Glavic et al., 2008 (case control)	30 cases with ELC and 30 controls	CCA, at 1.5 cm of bifurcation.	cIMT higher in patients with ELC than in controls (65.7 ± 12.2 *vs*. 65.4 ± 11.59 mm, respectively; p = 0.877).
Shrestha et al., 2009 (cross-sectional)	61 cases with ELC and 51 without ELC	CCA, 10 mm proximal to carotid bulb.	Patients with ELC had significantly higher cIMT than controls (0.90 ± 0.24 *vs.* 0.77 ± 0.15; *p* < 0.001)
Ziyrek et al., 2016 (case control)	65 cases with ELC and 65 controls	CCA, at 1 cm of bifurcation.	cIMT higher in ELC group than in controls (0.85 ± 0.16 *vs*. 0.60 ± 0.15; p < 0.0001). Independent association in regression analysis.
Rerkimitr, et al. 2017 (cross-sectional)	91 cases with anterior tragal crease (40% of them with ELC) and 56 without anterior tragal crease.	CCA, 10 mm proximal to carotid bulb.	cIMT higher in subjects with anterior tragal crease than in those without (0.79 ± 0.25 *vs*. 0.66 ± 0.10: no *p* value reported). In the ELC sub-group no association with cIMT was found.

ELC: earlobe crease; CCA: common carotid artery; cIMT: carotid intima-media thickness.

In summary, this study shows that the association between ELC and the cIMT is related to age. Further longitudinal studies are needed to determine whether the ELC is an innocent bystander, or if it could be considered a truthfully marker of carotid atherosclerosis and a stroke risk factor.

## Declarations

### Author contribution statement

Oscar H. Del Brutto: Conceived and designed the experiments; Wrote the paper.

Robertino M. Mera: Analyzed and interpreted the data; Contributed reagents, materials, analysis tools or data.

Aldo F. Costa: Performed the experiments; Analyzed and interpreted the data.

Pablo R. Castillo: Conceived and designed the experiments.

Gautam Matcha: Performed the experiments.

### Funding statement

This work was supported by Universidad Espíritu Santo – Ecuador

### Competing interest statement

The authors declare no conflict of interest.

### Additional information

No additional information is available for this paper.
